# A Theoretical and Practical Treatise on Midwifery, Including the Diseases of Pregnancy and Parturition

**Published:** 1850-04

**Authors:** 


Art. VII.—*A Theoretical and Practical Treatise on Midwifery, including the Diseases of Pregnancy and Parturition.* By P. Cazeaux, Adjunct Professor in the Faculty of Medicine of Paris, etc., etc. (Adopted by the Royal Council of Public Instruction.) Translated from the second French edition, with occasional notes and a copious index, by Robert P. Thomas, M. D. With one hundred and seventeen illustrations. Philadelphia: Lindsay and Blakiston. 1850.

Of the making of books there seems to be no end. Solomon complained of this tendency as one of the fixed facts of his day, when the mode of reproduction bore about the same relation to that of the present, that the movements of the ox do to the facilities of the magnetic telegraph. But there are consolations for the great extension of the business. Abernethy consoled himself for the immense increase of physicians in his day with the fact, that though physicians had greatly increased, diseases had increased in an equal ratio. Readers too have increased in a fair ratio with the increase of authors.

It is somewhat strange that upon a single department of practical medicine, that of obstetrics, a department consisting of many facts, and of a few principles, there should be such an endless variety of books. The literature of obstetrics is as prolific as though its exclusive province were to bring forth books, and we have every conceivable variety of presentation, monstrosities, malformations, abnormal as well as normal works, and not a few that show indubitable marks of having passed through minds of very small pelvic capacity.

We do earnestly wish that some one, well qualified for the work, would undertake to compress all the essential and valuable matters in obstetrics, contained in a world of books, into one readable volume. A more acceptable service to the accoucheur could scarcely be performed, and it would be, in every point of view a most practicable enterprise. The various works which command the attention and confidence of the profession have an immense deal of detail that is common to the whole class, and each authoritative work claims public regard on account of some excellence in comparatively confined particulars. Now we hold it to be a very practicable thing to take what is common, mere routine detail, in such works as those of Francis Ramsbotham, Baudalocque, Velpeau, Rigby, Collins, Churchill, Cazeaux, and as many others as any one may choose to consult, and array, in their proper places, the peculiar excellencies of each, and compress them into one tangible, manageable bale of reading matter. Commerce would be in a confused perplexity with bags of cotton, but the hydraulic press has removed all that kind of trouble. A discriminating medical mind, of hydraulic power, would confer no ordinary benefits upon medical readers by an effort at the compression of obstetrical literature. It would be something of value to be able to find promptly, all the recorded facts observed by obstetricians, and all the essential principles of the practice of obstetricity, in a portable form. The importance of this may be, in some degree, appreciated by reference to the subject of Dr. Hewett's interesting paper in the present number. In the case detailed in that paper, we have a malformation which, under almost all conceivable circumstances, must produce a fatal issue, after parturition. Yet, with the exception of Churchill, scarcely any popular author on obstetrics, even refers to such a case. It may be said that this proves the extreme rarity of this malformation, and while we admit the fact, we point to the rarity as a powerful reason why obstetrical teaching should warn practitioners against this danger. It is not the bold, jutting headland, the frowning cliff, nor the prominent reef that places the vessel in danger; the sunken, unknown rock, "not laid down in any chart," is the perilous one.

We give this merely as an illustration—examples might easily be multiplied to show the necessity of having every important particular, connected with obstetrics, gathered together in one volume, of easy access. There is no good reason why the excellencies of twenty good authors should be spread through twenty volumes, while it would be a possible thing to arrange them all in one.

These remarks are called forth by the necessities of the case, not in any spirit of fault-finding with the book before us. We, indeed, selected the occasion of having a work of the sterling excellence of this, for making the suggestions in the foregoing remarks. We preferred doing so over the work of Cazeaux rather than over such as may be easily found—those that are far inferior to it. In the multitudinous collection of works devoted to the propagation of human beings, and to the details of parturition, none, in our estimation bears any comparison to the work of Cazeaux in its entire perfectness, and if we were called upon to rely alone on one work upon accouchment, our choice would fall upon the book before us, without any kind of hesitation.

In all that constitutes a medical book of authority, in all that may aid inexperienced persons in perilous circumstances, in all that arms and equips an accoucheur for the performance of all his duties, we know of nothing superior to this work. The universal design of the author is to make himself a reliable pilot in every possible contingency, and his principles are uttered in the most lucid terms, and impressed in the most cogent manner. Take for instance his adoption of Nægèle's beautiful and all-sufficient simplification of Baudelocque's Chinese puzzle—the presentations. In Baudelocque's classification, we have one hundred and two distinct presentations enumerated; in the work before us this tangled web is reduced to thirty positions, and while the mind grasps these with ease, it is left free to gather other information, instead of cramming every chamber of memory with the refining of Baudalocque's presentations. This is but a solitary example of an excellence that reigns in every department of the work.

The original edition of this work has long been known to the profession, and wherever known has been highly prized. To those who are not familiar with its merits, we commend its invaluable chapters on embryology, a department of science in which the labors of Wagner, Kollicker, Leuckardt, Valentin, and Bischoff have effected a revolution quite equal to that made in electro-magnetism by the labors of Œrsted and the two Ampères. In our early medical studies, the philosophy of Generation bore no indistinct resemblance to Washington Irving's Ichabod Crane. It was loose, gangling, and perpetually ludicrous. Leuenhoeck's microscopic observations, looked upon then very much as Munchausen's works are by half grown men; Spallanzani's experiments with frogs, and Haighton's with rabbits, constituted the villages of the domain of the science; the country roads were travelled on the back of an animal that often fell by reason of the grievous loads it was made to carry—loads of such enormous burden, that one would suppose any beast of burden would have died under them, even if it had possessed a much larger share of vitality than our carrier ever did. The "ship of this medical desert," to which we allude, was named SYMPATHY. We were once in the habit of loading the animal ourself, and often wondered then, at a time when we *suspected* it of some vital power, how it moved under its loads. It has strayed away from medical science, and has ceased to be a carrier among philosophers. It was very patient and expansible, and is supposed to be in active employment among Mesmerizers, biologists, and fortune tellers. It feeds upon such provender, as those things furnish, with great greediness, but starves to death under the eye of observation, and the accumulation of truth. The labors of such men as Wagner, Leuckardt, and Bischoff were very fatal to the life of SYMPATHY, and under their assaults it made a seasonable retreat, and will never again be used by medical riders.

In traveling over the pleasant fields that have grown luxuriant crops in the last few years, we are often compelled to pause, and ask how such nonsensical stuff as sympathy ever retained its dominion over a well-informed mind, even for the space of a day. To those who love medical philosophy for its own sake, who delight to see truth springing up in its vigor and power, and who rejoice in the progress of medical science, we commend the subject of Generation, under the lucid pen of Cazeaux. We know of no more healthful recreation for a good mind than such reading.

It is difficult to single out points of excellence in a book like this, where all is of the highest quality, but we cannot refrain from impressing on the mind of our readers, on all suitable occasions, the admonitions contained in the following remarks of the American translator of this book He says: "The valuable experience derived from his [Cazeaux's] hospital practice, has clearly shown that the unaided powers of nature are quite sufficient to effect the expulsion of the child, in a vast number of those cases which have heretofore been considered as absolutely requiring the intervention of art; and his detailed observations, in the fourth part, on the subject of difficult labors, will afford another, and, I trust, a convincing proof of the truth of the maxim, that MEDDLESOME MIDWIFERY IS BAD." In this salutary hope we cheerfully join. We have an inexpressible horror of the use of instruments in midwifery, and feel more than an ordinary share of thankfulness in the fact, that, in a long practice of the art, we have never yet been compelled to resort to instruments—thus far, it has been our good fortune to succeed by an unfaltering faith in the powers of nature. The lessons of Cazeaux are confirmatory of our own observations, and to those who are inexperienced in obstetrical art, we commend the author of this work as one of the safest guides in the use of instruments that can be found.

The original work of M. Cazeaux was published in France upwards of ten years ago, and such were its merits, that in 1841, it was adopted by the ROYAL COUNCIL OF PUBLIC INSTRUCTION. Since that time a second edition has been published in France, and this occasion was seized upon by the author to enlarge the work. It was remodelled throughout, so that the second edition contains one-third more matter than the original one, and has a large addition of plates, illustrating the mechanism of labor, and "of the varions phenomena exhibited by the human ovule in its successive transformations." The work may now be regarded as an authoritative exhibition of the principles of obstetrical science, as taught and practiced in France at this time.

The translator, Dr. R. P. Thomas, deserves credit for the labor he has bestowed on this American edition. He has performed his duties in a very creditable style, and is entitled to the thanks of those who do not read French, for having enabled Cazeaux to address himself almost as well to American readers, as though the work had been written in the English language. We have met with but few translations that conveyed the spirit of the original with more complete success.

The publishers, too, have discharged their duties creditably. The illustrations are generally good, and the mechanical execution of the work is respectable.

We are indebted to Beckwith & Morton for our copy of this book.

